# High-Dose Probiotic Mix of *Lactobacillus* Spp, *Bifidobacterium* Spp, *Bacillus coagulans*, and *Saccharomyces boulardii* to Prevent Antibiotic-Associated Diarrhea in Adults: A Multicenter, Randomized, Double-Blind, Placebo-Controlled Trial (SPAADA)

**DOI:** 10.1093/ofid/ofaf316

**Published:** 2025-05-27

**Authors:** Cynthia Isabel Ortiz-Lopez, Adrian Camacho-Ortiz

**Affiliations:** Department of Infectious Diseases, Hospital Universitario Dr Jose Eleuterio Gonzalez, Monterrey, Nuevo Leon, Mexico; Department of Infectious Diseases, Hospital Universitario Dr Jose Eleuterio Gonzalez, Monterrey, Nuevo Leon, Mexico

To the  Editor—We were particularly interested in the recent publication by Hodzhev et al [1], which explores the use of a high-dose probiotic combination—comprising *Lactobacillus* spp, *Bifidobacterium* spp, *Bacillus coagulans*, and *Saccharomyces boulardii*—for the prevention of antibiotic-associated diarrhea (AAD) in adults.

The authors note the use of enterosolvent cellulose capsules for both the probiotic mix and the placebo. However, we observed that certain components, such as maltodextrin and magnesium stearate, were not mentioned. There are reasons to believe that the inclusion of these ingredients in the cellulose capsule could influence gut health outcomes.

Upon further investigation, we noted that maltodextrin, a carbohydrate derived from plant sources and converted into glucose [2], may have side effects. These include exacerbation of intestinal inflammation, reduced mucus production, and increased susceptibility to colitis, all of which appear to be dose dependent in murine models [3]. Additionally, it is important to differentiate among types of maltodextrin. Resistant maltodextrin, a type of resistant starch (type V), has been shown to cause abdominal discomfort or even diarrhea when consumed at high doses. This is due to its fermentation in the colon by gut bacteria, leading to the production of short-chain fatty acids such as acetate, propionate, and butyrate, which can cause bloating, flatulence, and abdominal pain. Moreover, this fermentation may increase osmotic pressure, potentially triggering diarrhea [4].

Magnesium stearate—commonly used in the formulation of dietary supplements, pharmaceutical tablets, capsules, and powders [5]—may have significant effects on gut health. Upon ingestion, magnesium stearate is metabolized into magnesium ions and stearic and palmitic acids. Stearic acid, a long-chain saturated fat, has been associated with a laxative effect, potentially leading to bowel spasms and, in some instances, allergic reactions [5, 6]. While manufacturers typically use magnesium stearate in small quantities, we believe that its potential impact on gut health warrants disclosure in the study.

In our opinion, there are notable discrepancies in the article regarding the role of antibiotics in the studied population, as the majority of participants were drawn from pulmonary and ear, nose, and throat clinics. While *Streptococcus pneumoniae* is a well-established cause of respiratory infections, these infections are more often attributed to viral pathogens, which generally do not require antibiotic treatment [7]. It would be valuable if the study provided further details on the bacterial pathogens identified, including whether they were confirmed by culture, and the diagnostics tests used to distinguish bacterial infections from viral ones.

Furthermore, the article provides insufficient detail on participant follow-up, especially regarding how treatment adherence and compliance were monitored. Additionally, any changes in participants' diets should be documented, as well as the use of other medications with potential gastrointestinal effects or possible interactions with the administration of antibiotics. For instance, tracking the long-term drugs most commonly used by participants, along with any prescribed antibiotics, could help identify potential confounding factors contributing to symptoms such as diarrhea.

The observed improvement in quality of life, as reflected by a reduction in the incidence of AAD with the probiotic mix as compared with placebo, may be a result of chance. While we commend Hodzhev et al for their investigation into the prevention of AAD in adults, further details are necessary to fully contextualize the reported findings and provide well-informed recommendations regarding the use of the high-dose probiotic mixture studied.

## Notes

***Author contributions*.** Both authors have seen and approved the letter.

***Financial support***. None.
